# Supplementary material for: Addressable nanoantennas with cleared hotspots for single-molecule detection on a portable smartphone microscope
Source: Nat Commun. 2021 Feb 11;12:950. doi: 10.1038/s41467-021-21238-9 (PMC7878865; doi:10.1038/s41467-021-21238-9)
Supplement: Supplementary file 1 — Supplementary Information [file 41467_2021_21238_MOESM1_ESM.pdf]

Supporting Information for

# Addressable Nanoantennas with Cleared Hotspots for Single-Molecule Detection on a Portable Smartphone Microscope

Kateryna Trofymchuk[1,2]†*, Viktorija Glembockyte[1]†*, Lennart Grabenhorst[1], Florian Steiner[1], Carolin Vietz[2], Cindy Close[1], Martina Pfeiffer[1], Lars Richter[2], Max L. Schütte[2], Florian Selbach[1], Renukka Yaadav[1], Jonas Zähringer[1], Qingshan Wei[3], Aydogan Ozcan[4], Birka Lalkens[5], Guillermo P. Acuna[6]*, and Philip Tinnefeld[1]*

[1] Department of Chemistry and Center for NanoScience, Ludwig-Maximilians-Universität München, Butenandtstr. 5-13, 81377 München, Germany
[2] Institute for Physical and Theoretical Chemistry - NanoBioScience and Braunschweig Integrated Centre of Systems Biology (BRICS), Technische Universität Braunschweig, Braunschweig, Germany
[3] Department of Chemical and Biomolecular Engineering, North Carolina State University, Raleigh, NC, 27695, Unites States
[4] Electrical & Computer Engineering Department, Bioengineering Department, California NanoSystems Institute (CNSI), and Department of Surgery, University of California, Los Angeles, California 90095, United States
[5] Institut für Halbleitertechnik, Laboratory for Emerging Nanometrology LENA, TU Braunschweig, Langer Kamp 6a/b, 38106 Braunschweig, Germany
[6] Département de Physique - Photonic Nanosystems, Université de Fribourg - Faculté des Sciences et Médicine Chemin de Musée 3, CH-1700 Fribourg, Switzerland

† These authors contributed equally: Kateryna Trofymchuk, Viktorija Glembockyte

***** email: kateryna.trofymchuk@cup.lmu.de; viktorija.glembockyte@cup.lmu.de; guillermo.acuna@unifr.ch; philip.tinnefeld@cup.uni-muenchen.de

# Table of contents


Supplementary Table 1. Temperature ramp used for folding DNA origami nanostructures ........................................ 3

Supplementary Table 2. Unmodified staple strands used to fold the DNA origami nanostructures .............................. 4

Supplementary Table 3. Modified staple strands used for the immobilization of the DNA origami structure, nanoparticle binding and fluorescence labelling ................................................................................................ 9

Supplementary Table 4. Modified staple strands used for the sandwich detection assay ........................................... 10

Supplementary Table 5. Modified staple strands used for solution synthesis of NACHOS ....................................... 11

Supplementary Figure 1. Base layout of the DNA origami nanostructure used to build NACHOS ............................ 12

Supplementary Figure 2. Staple layout of the DNA origami nanostructure used to build NACHOS .......................... 13

Supplementary Figure 3. Exemplary single-molecule fluorescence transients of Alexa Fluor 647 dye ....................... 14

Supplementary Figure 4. Fluorescence scans of the DNA origami reference structure aquired in buffer solution ...... 15

Supplementary Figure 5. Incubation time series for the reference DNA origami structure and the NACHOS ........... 16

Supplementary Figure 6. Testing specificity of binding for variations of the Oxa-48 DNA sequence ........................ 17

Supplementary Figure 7. Exemplary fluorescence transients of the sandwich assay in DNA origami reference structures without nanoparticles and in NACHOS ................................................................................................ 18

Supplementary Figure 8. Fluorescence scans of the DNA origami reference structure acquired in blood serum ........ 19

Supplementary Figure 9. Additional fluorescence transients of single Alexa Fluor 647 ............................................ 20

Supplementary Figure 10. Fluorescence transients of single ATTO647N dyes in NACHOS recorded on the smartphone microscope ................................................................................................ 21

Supplementary Figure 11. Negative controls on the smartphone ................................................................ 22

Supplementary Figure 12. Additional fluorescence transients of the sandwich assay in NACHOS measured in buffer solution ................................................................................................ 23

Supplementary Figure 13. Additional fluorescence transients of the sandwich assay inside NACHOS measured in blood serum ................................................................................................ 24

Supplementary Figure 14. Bleaching step analysis ................................................................................................ 25

Supplementary Figure 15. Fluorescence enhancement histograms of a single ATTO 647N dye in NACHOS of a previous design ................................................................................................ 26

Supplementary Note 1. Discussion pertaining the costs of the smartphone microscope ............................................ 27

Supplementary Note 2. Discussion pertaining the costs per sample for the diagnostic assay on a smartphone microscope. ................................................................................................ 28

Supplementary References ................................................................................................ 28

**Supplementary Table 1. Temperature ramp used for folding DNA origami nanostructures**

| Temperature [°C] | Time [s] |
|---|---|
| 65 | 300 |
| 65 | 50 |
| 64 | 95 |
| 63 | 95 |
| 62 | 95 |
| 61 | 95 |
| 60 | 540 |
| 59 | 540 |
| 58 | 1140 |
| 57 | 1740 |
| 56 | 2340 |
| 55 | 2940 |
| 54 | 3540 |
| 53 | 3540 |
| 52 | 3540 |
| 51 | 3540 |
| 50 | 3540 |
| 49 | 3540 |
| 48 | 3540 |
| 47 | 3540 |
| 46 | 3540 |
| 45 | 3540 |
| 44 | 2940 |
| 43 | 2340 |
| 42 | 1740 |
| 41 | 1140 |
| 40 | 1140 |
| 39 | 1140 |
| 38 | 540 |
| 37 | 540 |
| 36 | 290 |
| 35 | 290 |
| 34 | 290 |
| 33 | 290 |
| 32 | 290 |
| 31 | 290 |
| 30 | 290 |
| 29 | 50 |
| 28 | 50 |
| 27 | 50 |
| 26 | 50 |
| 25 | 50 |

**Supplementary Table 2. Unmodified staple strands used to fold the DNA origami nanostructures**

| Name | Sequence (5'→3') |
|---|---|
| 1 | TTTAAATGTTTGCTGAGATTTAGGACCCACGCGAA |
| 2 | TTAGAACGCAATTAAGACAAATACATACATAAA |
| 3 | TTTAAGCAAATTCACAAAGTATTAAGAGGCTCGGA |
| 4 | TAAATACCCGGATATCATCAACGGTCAATCATAAGACCATCGATAC |
| 5 | GAAGGGATAGCGAGATAGTTCCGGCCAGGAAGAAGAATGAGGT |
| 6 | GCAACTGGCGAAAGGGGAGTAAAGTTGCCGGAGTGAGACCGGTCCAAAC |
| 7 | ACGAGGAGAGGCGGTTTGATGGTGGGGCCCACCCT |
| 8 | CGGTGTACAGACCAACAAAGCTAACGGAAAAAATCTACG |
| 9 | AATATCGGCACGCGCGGGCCGGAAGCATAAAAGCT |
| 10 | CAGAACAATATATCGGCCATCAAACACAGTTGAAAGGAA |
| 11 | TGAGGAAAACAGCCTGATTGCTTTGTTGC |
| 12 | GAACGCCTCCATATTATTTTA |
| 13 | AGTTCTGTCCCCCCCGAGGCGCTGGCAAGTGTTTG |
| 14 | CTTAAATCCCGGCGGTTGTG |
| 15 | AGCAATACTTCATCACGCAAATATCGCCAGTA |
| 16 | TTCATTTACCATATTGCGGAACAAAGAA |
| 17 | CTACAATTTTTTTGAAGAAAAAGCTTTAAAACAGAAATAAAGAAAAAT |
| 18 | CCTACATATCTAAAGCATCACCTCAAATTTGC |
| 19 | GGTGGCTCCAACGGCATTTCGCACTCAATCCACGCCATCCA |
| 20 | CGGAATTACCGTGTCGCAAGACAAAGAAAACAGTAAACAAAC |
| 21 | TTTCAATGATAAATTAATGC |
| 22 | GTCGAGGACCCGCCGCACCTTTTACATCCGCTGAGCAT |
| 23 | GTAATCAGAAACGAGCCTTTAGTGCCTTCTCAGAACGA |
| 24 | GCGACCCACCAAGTAGAATCATTAAAGGTGAAAATA |
| 25 | GTCTGAGCAAAAGAAGATAATGGGAAGGAG |
| 26 | TCACGCGTGGGAACAAATGTCACTGCGCGCCGCGG |
| 27 | ATTAGAGCATTTTTGCGAGCTGAAAAGGTCTA |
| 28 | TGTGATAAATTTAGCCGGAACGAGATATATTCTCA |
| 29 | TCCCGGGCGAAAGCCACCGTCTTTCCAGAGCCGAA |
| 30 | AATAAACCAGAATCTTTTCATAATCAGGA |
| 31 | CAGACCAGTTACAAAATAAAGGCTTCAGTAGGAGTATTATTAATGC |
| 32 | CGTAGGCGCATAACTGACCAACTTTGTTGCGCGATACATTGCAAAAG |
| 33 | AATAATAACCGGCGCAGAGAGTAATCTCGCCT |
| 34 | CATTATATTTTATCTTCTGACCTAAAGATGATCAATATA |
| 35 | AGGACGTTAAGAACGGTTTAATTTCAACGAGAAACCAA |
| 36 | AGGAGGCTTTAACGCCAAACGAACTGCTCAT |
| 37 | ACCACCCTTAGATGAGTGACCTGTCGTGCCAGAAT |
| 38 | GGTGATAAGAACTGGCATGATAATAACAGCCCTTTAATATC |
| 39 | CCCCTTTTCTTGTGTGAAATTGTTAAAGCACTTGT |
| 40 | CATTTAAACTCCATATAGATTCATCAGTGAACAAGAAACTCATC |
| 41 | AACAGACAATAGTTTATCCGCTGGTAAATGTGCAG |
| 42 | CGGATCGGATGTGCTGCAAGGCGATCAGTGCCAGGTGGAGCC |
| 43 | CCGAGCTCGAACTTGACGAAAGGTAAGAGGCATTTATTT |
| 44 | TGGGCACTAAAAAAGAGTCTGTCCTTTGATTTCAAACTTAC |

| | |
|---|---|
| 45 | GAGTCAACTAATTTAGGCAAGTAATCCTGAACAGA |
| 46 | AGAGTTCGTAAAGCTGATCTCATAAGGATTGACTGCCAGTTTGAGGCAG |
| 47 | TACGCGGGATACGAGGGCAACGGAATTATACCAAG |
| 48 | ATCCTTTGCAACAGGAAAAACGCT |
| 49 | GAAGGTATTATCACCCAGCAAAATCACCTTACCATTAGC |
| 50 | TTGCAAAGACAAAAGGGAATGAAATAGCAAGCAGCACC |
| 51 | GCAAGACTGGATAGCGTGAATCCCCTGTATGCGC |
| 52 | AGCACCCTCAAATCCTCCAGGAAGGGTCATTCCTTTAATTGTACAGGTG |
| 53 | TTTGCGTATTGACAATTCCACACAAAATTGGG |
| 54 | AAACGGACGACGTCGGTGACGCAACAGCGAGTATAGTTATTTTGATGGGG |
| 55 | ATATAATACACGTACTACACCAGCTAACACCATTCACCAGTCACA |
| 56 | TATTTTAACCTCAAAAGCTGCATTGCCTGGGGTGCCTAAATCCTTAGAC |
| 57 | AAAGGAAGCTTGATGTTGAAACCTG |
| 58 | GTCAGACCTCAAGAGAAGGAT |
| 59 | TTATCAGCTTGCTTACACTAT |
| 60 | AAAAATTAAAGCCTATTATTCTGAAGTTGATAGATTGCAAACCCTC |
| 61 | TTTGCGGGCCTCTGTGGTGCT |
| 62 | CACCGGAATCATTTCAAAATTATTT |
| 63 | TAAAGGAAGCTCTGGAACTGCGAACGAGTAGGCATAAACTGTAATGTCA |
| 64 | GAGCGTCCACTACCTCCGTAATTTTAGTTACAAAATCGCCGT |
| 65 | TACCAGAATCAAGTTTGCCTTATTTAAAAACTAATAAGACCGCCATGC |
| 66 | GCAGCAGAGGTCGTCGCAATTGCG |
| 67 | TGAGATCGGCTATAATATACCGACAGGGAAAGAGCGAAAGGAGCGGCAGT |
| 68 | CTTGGTAACGCCAGGGTACGACGTGGAT |
| 69 | CGCGCAGTATATTCGACAATGAATATACAGTA |
| 70 | AAGAGGTAGTACCTTGAGAAAGGCCGGACAATGCCATAGTAG |
| 71 | TGCACGACAATTGCGAATGCCCCCTCGGCTGGCCA |
| 72 | GCTTTGAGGACTAATACGAAGAAAACGAAAGAGGCCCCAGCGGATT |
| 73 | ATATAAAATTCATATGGTTTATTACCGAGGAA |
| 74 | GCAGTTGGTAAAAAGGCGGCCGCGTGGTGGGTGGTAGCAGGCTGCA |
| 75 | GTCCTTTCATGCATGTCCCAGTAAAGTGCCCGTATAAAAGGAGGTAATC |
| 76 | ACATTACAAAGGATTAAGGTGCCGTCGAGAGGACATGAAACAA |
| 77 | TAGTACTAAAGTACGGTGCCGAAAGATTTTTGATTGTAATTTTGTTGGGT |
| 78 | AGTGAATTTTCCTCAAACCCTCAGAGCCACCGAACCCACAC |
| 79 | TTATTCGGTCGGGTATTAGCCGTTTTTTCGATTTA |
| 80 | TCATCGTAACATTCCAAGAACATAGCCCCCT |
| 81 | GCCGCTACCACCACTGCCGTATCCGCTCGGCGCCAGCTGGTC |
| 82 | ACAGTGCTTTACCGAACGAACTGGTTGCTAGCGGTAAC |
| 83 | TGCCCGCTTTCCAGGTGTTGTTC |
| 84 | ATAGAGCCGCACTCCAAGTC |
| 85 | GCGGTCAGTATAGAAGATTAGCCCTTAAAGGGATTTTAG |
| 86 | GGGGTTTATATCGCATATGCATTGACCATTAGATA |
| 87 | ATTCTAGCGATGTGTAAAAATGAATCGGCCAAAAA |
| 88 | AAGTTTTGACGCTCAAATCCGGTATTCTAATAA |
| 89 | TACTGTGTCGAAATCCGCAAAGTATAGCAAC |
| 90 | TATTAAATCATACAAAATCATAGCGTCAAATTAT |
| 91 | CACGGGGGTAATAGTAAAACAGTTAGACGTTAGCCCTCAACAACCCAG |

| | |
|---|---|
| 92 | GACACGTAGATCCTTATTACG |
| 93 | ACCAACATGGCGCGTAACGATCTTACAACATTTTG |
| 94 | TTAAAGAGATCTATGACCGCTAAATCGGTTGTCCC |
| 95 | AAAAGAATTTCTTAAACATTACGAGACCAAAA |
| 96 | CCTAGTTTCCTTTCACCACTTGTAGCAGCACCGACAGTATCGGCCTACCG |
| 97 | CTGTCATACCGGCCCTGGCCCTGAGAAGA |
| 98 | AACTGTAAAACGACGGCTAAGTTGCGC |
| 99 | AAAGTCTTTCCTTATAAGAGTGTACACAGACAGTAAATGAG |
| 100 | GCAAACCACGGTTTTGTCACAATCAAAAGTAACCG |
| 101 | CATTGAAGACAGTTCATGAGGAAGTTGGGTAAATAC |
| 102 | AATTGTTTCATTCCATATTCAAAAAGCTATCAATTG |
| 103 | AGAGAGAAATAACAAGCGTTTGCCATAAGTA |
| 104 | TCAATGCTCAGTACCAGGGAGACTCGATTGGCCCA |
| 105 | ACCTTATGCGATTTTGGGAAGACAACATTAA |
| 106 | TAGTATCAAATTCTTACAGGCGTTTTAGCGAAACG |
| 107 | AGCGGGAGCTAAACAGGAGTTTTTACAATAGATTT |
| 108 | ACGGAGCCGTTAATCAGTGAGGCCTTG |
| 109 | TTTGACCGCCAGGAAAGCTAATCAGAGCAAACAAA |
| 110 | AGGAAGCGCAGCGATCCCGTGCCGCCGGAACGTAAACGATGCTGATACG |
| 111 | AGGACGTCAGACTGTAGC |
| 112 | ACTGTATCACCGTACTCCAGTTAACTGAATTCCGCCACTACGTGAAAATC |
| 113 | GAAAATTCGCAGGCGCTCAGATGCCGGGTTAATCTCCAAAGAGAACCTG |
| 114 | TCGCCGGCTGGAGGTTTCTTTGCTCACTTTTGGGTAGCTACT |
| 115 | CGACACGCCAAATTACCGCGCCCAAAATCCAAGCC |
| 116 | CAGAGCGGGGTCATTGCGTCTGGCCGGTTGAGCAGTCTTGCCCCC |
| 117 | TCCCATGCGTTCTTTGCCGATTTTCAGGTTTACGG |
| 118 | TAAAAGGAATGGCTATTAGTCGAACTGAAAAA |
| 119 | TCAGTGAGAATCAAATCAGATATAGAACAGCCCTCAGAGTACCGTTAATC |
| 120 | CTATGAGTAATGTGTAGAAAAGGGTTAA |
| 121 | AGACCGGCAAACGCGGTCCGTTTT |
| 122 | GGACAAATCACCTCAATATGAAAATTTGACGCTCA |
| 123 | TTTGACCAAAAGAAATACGTAATGCCACAGACTTTCATC |
| 124 | AAAAATAGGAGCCGGGCTCAGCAAATCGTTAAAAGGAGGCC |
| 125 | AATCAAGAATTGAGTTAAATAGCATTTTTTGTTATCCCTAGCAAGCGCC |
| 126 | GAATTGCCAGAATTCAACTATTACACCCAAATACCAGAACGAGTAG |
| 127 | GTTGCGTCGGATTCTCGTAGCATTCCTCGTAA |
| 128 | AGCCAACGTGGCACCAGAATCTTACCAACGCTACC |
| 129 | GCCACGAAACGTTCGCCACGTGCATCCGTAATGGGATAGGGCC |
| 130 | ATCCTGAAAACAAACCTTTTTTAATGGACGCGAGAGGTTTGA |
| 131 | TGCCTATAATAGGTATTATAGGATAAAAGCATAGTAAGAGCATCGA |
| 132 | ATCAAGATTGTTTGTATTCCTGATTATCATTTAATAAACTTT |
| 133 | CAAGGGGCAACTCATGGTCATAGCTAAGGGAGAGA |
| 134 | ACCGAGGCTGGCTGACCTTTCATTAGGTAGAAACCAGTC |
| 135 | GAGAACAAGCAAAACCAAATCAATATTTCGTCACTACAAGGATTTT |
| 136 | TTTGGACATTCTGGCCAATTGGCAGGCCTGCA |
| 137 | TGTACGGAGGGAAGTGAGCGCTTTAAGAATAGAAAAGAAACGCAAA |
| 138 | TACGTATCATGACTTGCGGGAGGTATCCTGAACCACCACTTGATATAT |

| | |
|---|---|
| 139 | ACGGAACGTCATTTAGTGATGAAGGCATAAAACTGGTGCCCCGGAA |
| 140 | GCAGCAACAATATCGAAGAACAGTAATAACATCACACC |
| 141 | GAGGGAATCCTGAGAAGTGGCCGATAAAACATATT |
| 142 | AAAACCGCCACCCTCAGATTTTAACGATACAGTCACCGGGATA |
| 143 | GTTTACCAGACGACTCAGAAGAGTCTGGAAAAGCCCAAA |
| 144 | AGACAATCGCCATTAAAAAAGAATCAGCAGA |
| 145 | TAGCGAGTCTTTACTCGATGATGTACCCCTTCCTGCTG |
| 146 | ATAACGGTAATTTTCACACCGATAGAAAGAG |
| 147 | TTCAAATTGAATTAATTAATT |
| 148 | GTACGAACGTTATTAATCTGTTTACTTTTTAATTAAAGCGA |
| 149 | TGTGCGGTTGCGGTATGCTCA |
| 150 | AGGCTTGCCCTGACTTTAATC |
| 151 | TGCTTCTGTAAACGAATTA |
| 152 | ATCTAGCCAGCAGCATCCCAGCGGTGCCGGTAATAATTTCGTAAA |
| 153 | AAGTTTGACCATAACAAAGTTTTGTCGAAGGAATGACAACAGGA |
| 154 | GGACGTCACCCGGTCGCAGTTTCATGTGCACGTTT |
| 155 | AATCAAATTAGTACCGCCACCGAGTAACGCGTCATCCGGAACCGCGCCTAAC |
| 156 | CGGAGAGCGGGAGAAATAAAGCCTCAGAATT |
| 157 | ACAGTGCGACTTTACAAACAAAAGCCAAGTCAATACTATCATTTCC |
| 158 | TACATCAAACTGAAAAAGAGACGCATACCAGTCGG |
| 159 | CGTGTGAATTATTAAGAGGGAGAAACAATAAACGTCAGACTCG |
| 160 | ACTAAATGGGCTTGAGATTGGCT |
| 161 | TGAGCAAAGCGTAAGTATAGCCCGGTTCGGAACCAGAATCCCTCAGAAAC |
| 162 | TCACAGAGAGTAACCCAAGCTATCCCAGCGCACGGAAATTGCAAC |
| 163 | ATACAGAACCCTTCTGACGTCTGAAAGAGCCA |
| 164 | GATAAAATCAGAGCCGGGACATCCCTTACACTAAA |
| 165 | CGCCAGCCAGAAAGCGTACTGAGTATGGTGCT |
| 166 | ATCCATGTAATAGATTAAGCACGTATAACGTGCGCTAGTTT |
| 167 | CATAACAGTTGATTACTCGGT |
| 168 | AACAAAATCGGCACGCTGCGCGTAACAGGGCGTTT |
| 169 | TGAAAGCCCAAAAGAAACCGACATTAGGGAGG |
| 170 | CCAGAGCGCCATACAGCGCCATGTTGATTCAGAAGCTAACAG |
| 171 | TTCCTCGCACGCTGATGGATTATTTACACAGAGATGTGGCAC |
| 172 | CTTAGCATCAGACGATCCACAACTATCTTTCCCAG |
| 173 | TACGCCAATTTAGAGCTTAATCTCACCCACCATAAGAAA |
| 174 | TATTTGCCGTTGCACATCTGCCCTTCACCGGTGTA |
| 175 | ACCATCGATAGGCCGGAAATTAGAGCGTCACCGACT |
| 176 | TTTAGAACCCTCATATATTTTAAATGGACAGTCGGTCAGG |
| 177 | TAGCATTTTGGGGCGCGGATGGCTTAGATCCAACA |
| 178 | AGCAAACGCTTAATAGCTATATTTTCATAACATCCAATA |
| 179 | TAATTACTAGCCTTAAATCAAGATTTTGCACAGCATTGGAGGCAG |
| 180 | TGATCGGGAAAGCTAACTCACATTTATTAATGCTTAGGTTG |
| 181 | GAAAGGAAGGGAAGAACCGGCGATCCCCGGCCGTGAGAGCCTCCGTCACGT |
| 182 | GAAGGTTATCTAAAAT |
| 183 | AAGGCCGCTTTTTGCG |
| 184 | CACCCTGAACAAGCCG |
| 185 | CTCGTCGCTGGCCCTCCTCCGTGCCTTAATTTAGAAACCAGTAC |

| | |
|---|---|
| 186 | TTTGGAACAAGACGCCGCCCCAG |

**Supplementary Table 3. Modified staple strands used for the immobilization of the DNA origami structure (biotinX), nanoparticle binding (npbindX) and fluorescence labelling.**

| Name | Sequence (5'→3') |
|---|---|
| biotin1 | biotin - AGAATATAAAGTCCCATCCGTTCTTCGGGG |
| biotin2 | biotin - AGTTACCAGAAGGAAAGCAGATAAGTCAGAGGGTAATCGCA |
| biotin3 | biotin - ACAACTTTCAACTGAGGCTATGT |
| biotin4 | biotin - AGGGCGATCGGTGCGGTGCGCAACCGGAAACAATCGGCGGG |
| biotin5 | biotin - TTCATCGGCATTGACGGGACCAATAGACCCTCAATTCATTCCAA |
| biotin6 | biotin - TAGATGGGCGCATCGTAACTTCAGGCGCCT |
| npbind1 | CATTTCGTCAACATGTTTTAAGTTTTAATTCGAGAAAAAAAAAAAA AAAAAAAA |
| npbind2 | GGTTATATAACTATATGTGAATAAAAAAAAAAAAAAAAAAAAAAAA |
| npbind3 | ACCATCAACCGTTCTAGCCGCAAAAAAAAAAAAAAAAAAAAAAAA |
| npbind4 | ATAAAAATGCTGATGCAATGTGAAAAAAAAAAAAAAAAAAAAAAA |
| npbind5 | AAAGAATTAGCAAAATTAAGCAGCCTTTAAAAAAAAAAAAAAAAAAAAAA |
| npbind6 | ACCACCAAAGGGTTAGAACCTCAATTACGAATAACCTAAAAAAAAAAAAAAAAA AAA |
| npbind7 | AATCATACAGCCTGTTTTGCTGAATATAATGCGAAAAAAAAAAAAAAAAAAAAA |
| npbind8 | AATATAATCCAATGATAAATAAGGCGTTAAAAAAAAAAAAAAAAAAAAAA |
| npbind9 | AAATCACCATCAATATGATATGACCGGAAAAAAAAAAAAAAAAAAAAA |
| npbind10 | CTTCAAAGCTGTAGCCAAATGGTCAATAAGCAAGGCATAAAAATTAAAAAAAAA AAAAAAAAAAA |
| npbind11 | AAAAGTTTGAGTAACATTATCAAAAAAAAAAAAAAAAAAAAA |
| npbind12 | AATACCGATCATCAGATTATACTTCTGAATGATGACATAAATCAAAAAAAAAAAA AAAAAAAA |
| base_dye ATTO542 | TTTGTGATCTCACGTAAATTTCTGCTCA-ATTO542 |
| hotspot_dye ATTO647N | TAATCACTGTTGCCCTGATTAAATACGTTAATA-ATTO647N |
| hotspot_dye AlexaFluor 647 | TAATCACTGTTGCCCTGATTAAATACGTTAATA-AlexaFluor647 |

**Supplementary Table 4. Modified staple strands used for the sandwich detection assay: 3 capture staples (captureX), synthetic 34 nt target strand (target34) and Alexa Fluor 647 imager strand (Alexa647 imager). Complementary regions are depicted in the same colour. The unmodified staple strands from Supplementary Table 2 and modified staple strands from Supplementary Table 3 which are replaced by the capture strands and therefore should be left in order to fabricate the NACHOS out are indicated in the second column.**

| **Name** | **Strands to leave out** | **Sequence (5'→3')** |
|---|---|---|
| capture1 | hotspot_dye strand from Table3 | TAATCACTGTTGCCCTGATTAAATACGTTAATATTTTTCGG GCAATGTAGACA |
| capture2 | 186 from Table 2 | TTCGGGCAATGTAGACATTTGGAACAAGACGCCGCCCCAG |
| capture3 | 156 from Table 2 | TTCGGGCAATGTAGACACGGAGAGCGGGAGAAATAAAGCC TCAGAATT |
| target34 | | TGTCTACATTGCCCGAAATGTCCTCATTACCATA |
| Alexa647 imager | | TATGGTAATGAGGACAT-AlexaFluor647 |

**Supplementary Table 5. Modified staple strands used for solution synthesis of NACHOS. Overhang modifications (modificationX) exchange biotinX staples from the Supplementary Table 3 of the DNA origami structure. Complementary regions are depicted in the same colour. Corresponding unmodified strands from Supplementary Table 2 and modified strands from Supplementary Table 3 should be left out.**

| **Name** | **Replacing strand** | **Sequence (5'→3')** |
|---|---|---|
| modification1 | biotin1 Table 3 | GTGATGTAGGTGGTAGAGGAAAGAATATAAAGTCCCATCCGTTCTTCGGGG |
| modification2 | biotin2 Table 3 | AGTTACCAGAAGGAAAGCAGATAAGTCAGAGGGTAATCGCA |
| modification3 | biotin3 Table 3 | GTGATGTAGGTGGTAGAGGAAACAACTTTCAACTGAGGCTATGT |
| modification4 | biotin4 Table 3 | AGGGCGATCGGTGCGGTGCGCAACCGGAAACAATCGGCGGG |
| modification5 | biotin5 Table 3 | TTCATCGGCATTGACGGGACCAATAGACCCTCAATTCATTCCAA |
| modification6 | biotin6 Table 3 | GTGATGTAGGTGGTAGAGGAATAGATGGGCGCATCGTAACTTCAGGCGCCT |
| mag1 | | TCTCCATGTCACTTCTTCCTCTACCACCTACATCACCTTCTTCTTCTTCTT - biotin |
| mag2 | | GTGATGTAGGTGGTAGAGGAA |
| mag3 | | AAGAAGAAGAAGGTGATGTAGGTGGTAGAGGAAGAAGTGACATGGAGA |

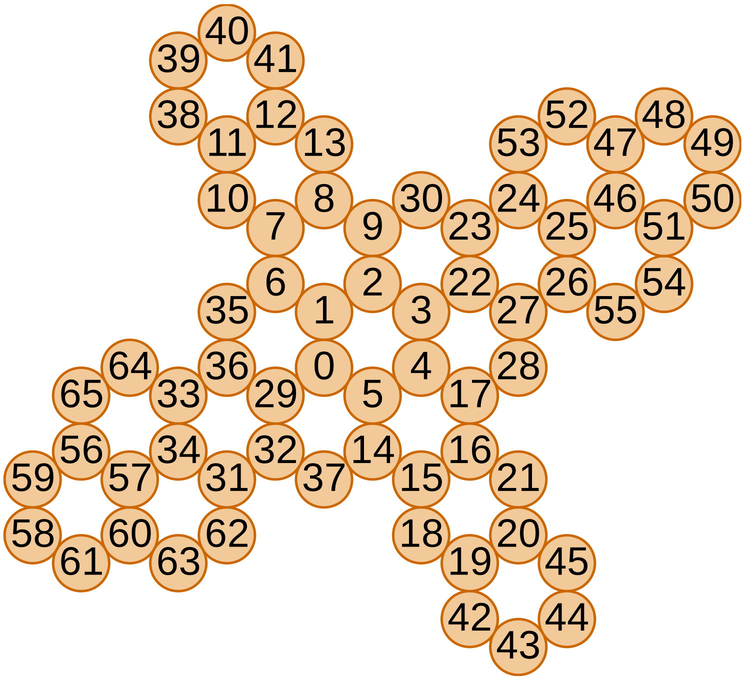


**Supplementary Figure 1. Base layout of the DNA origami nanostructure used to build NACHOS**

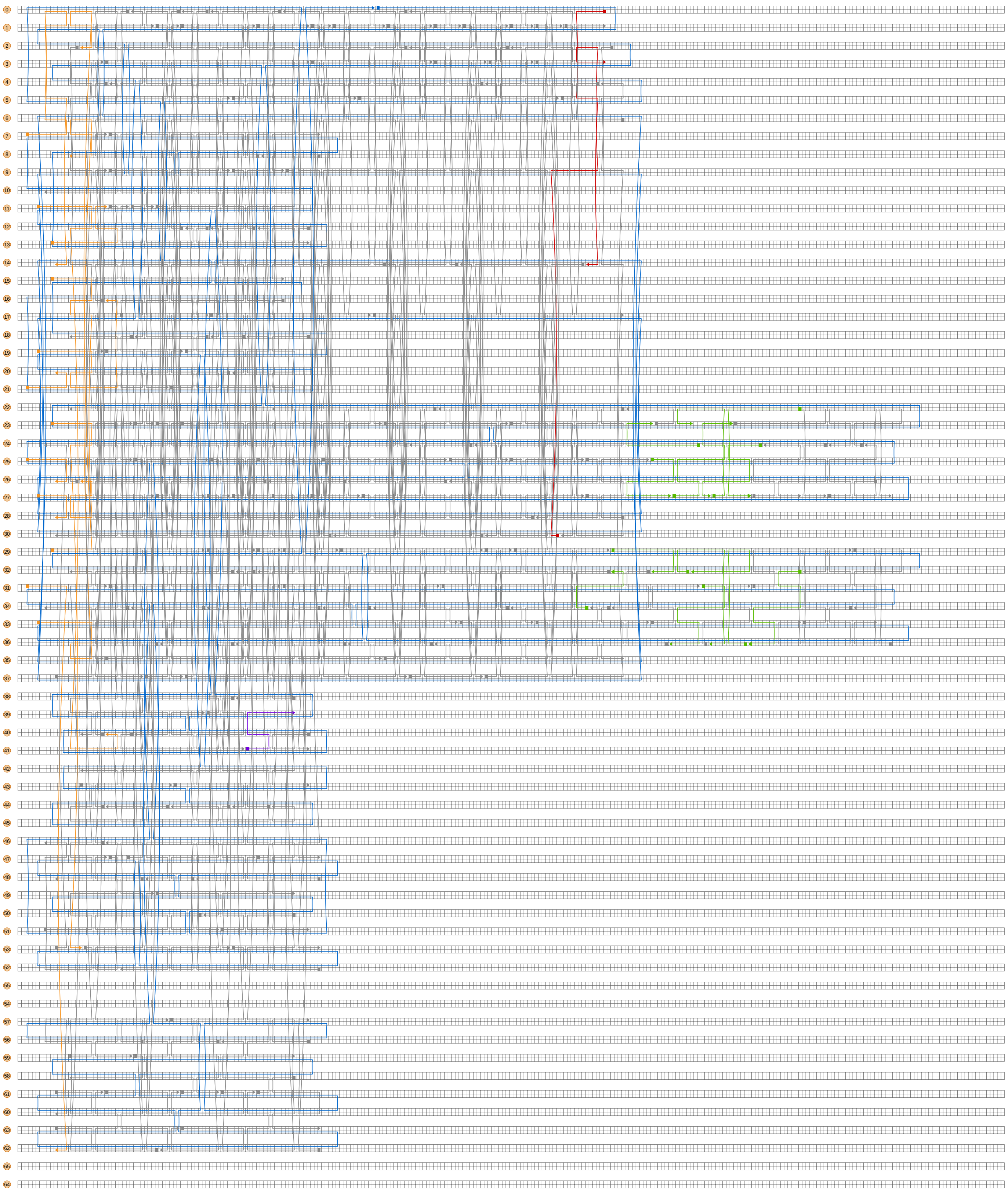

**Supplementary Figure 2. Staple layout of the DNA origami nanostructure used to build NACHOS (yellow = biotin staples, red = hotspot staple, green= nanoparticle binding staples, purple = base dye staple)**

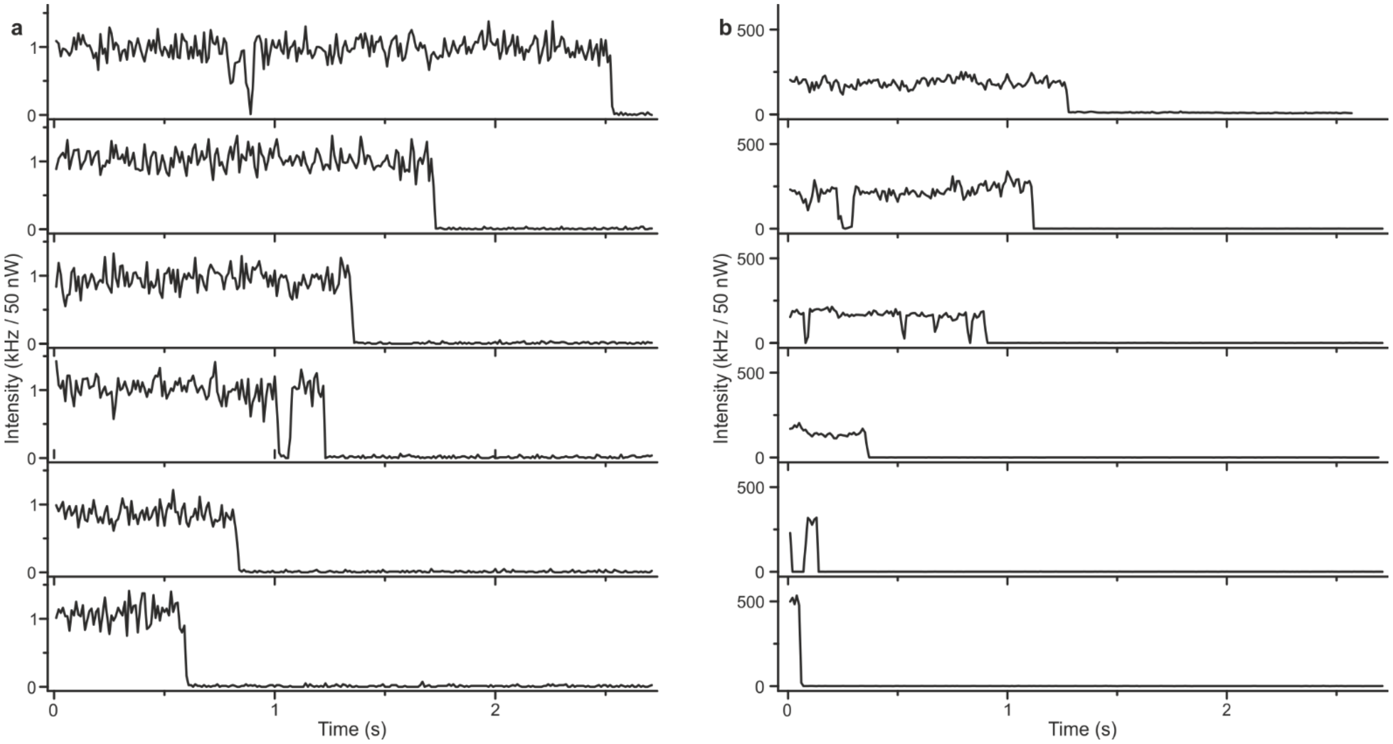


**Supplementary Figure 3. Exemplary single-molecule fluorescence transients of Alexa Fluor 647 dye in DNA origami reference structures without nanoparticles (a) and in NACHOS (b). The samples are measured at 639 nm with 200 nW and 50 nW excitation power for panel (a) and (b), respectively, and the transients are normalized to the same excitation power.**

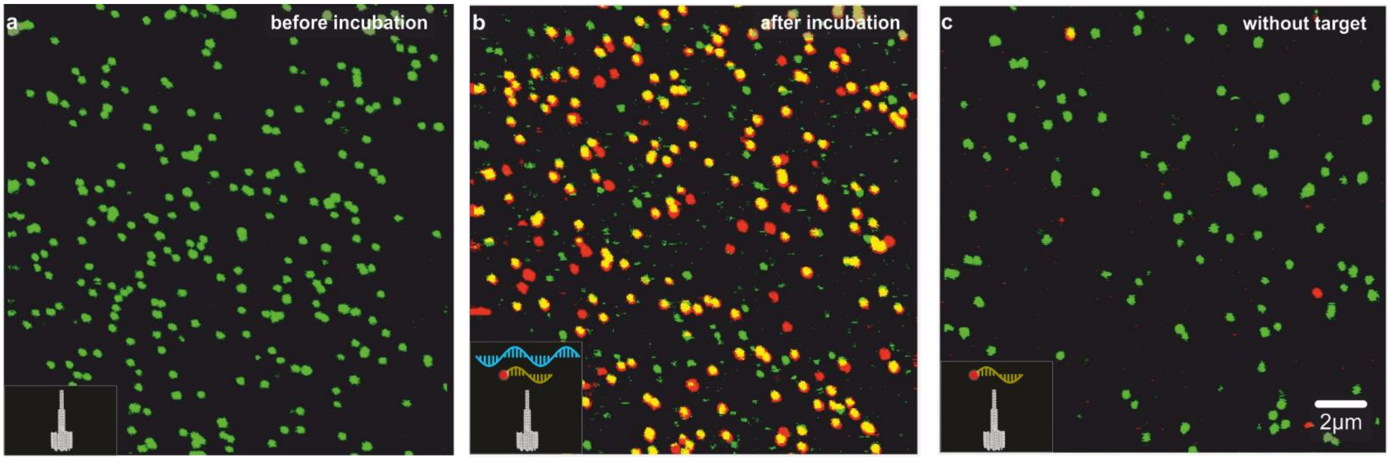


**Supplementary Figure 4. Fluorescence scans of the DNA origami reference structure (without nanoparticles) measured in buffer solution acquired before incubation (a), after incubation with the full sandwich assay (b), and after incubation with the imager strand only (c). Excitation was carried out at 532 nm and 639 nm with 2 µW excitation power. At least 20 different areas were measured for each sample.**

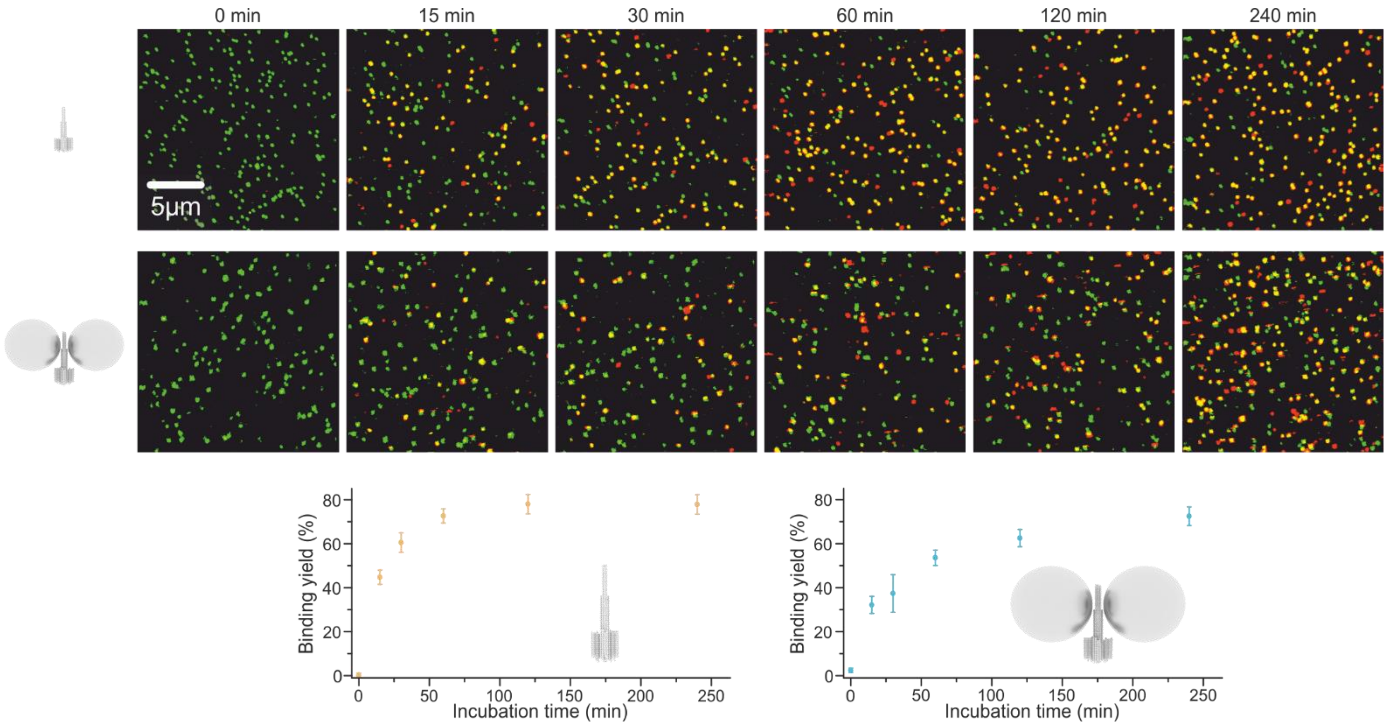


**Supplementary Figure 5. Incubation time series for the reference DNA origami structure (first row) and the NACHOS (second row). The binding yield efficiency for every incubation time is calculated from at least 4 different areas of the sample and represented at the bottom. The error bars represent the standard deviation from the mean. Measured at 532 nm and 639 nm with 2 µW excitation power for the reference structure and at 532 nm with 2 µW and 639 nm with 500 nW for the NACHOS structure.**

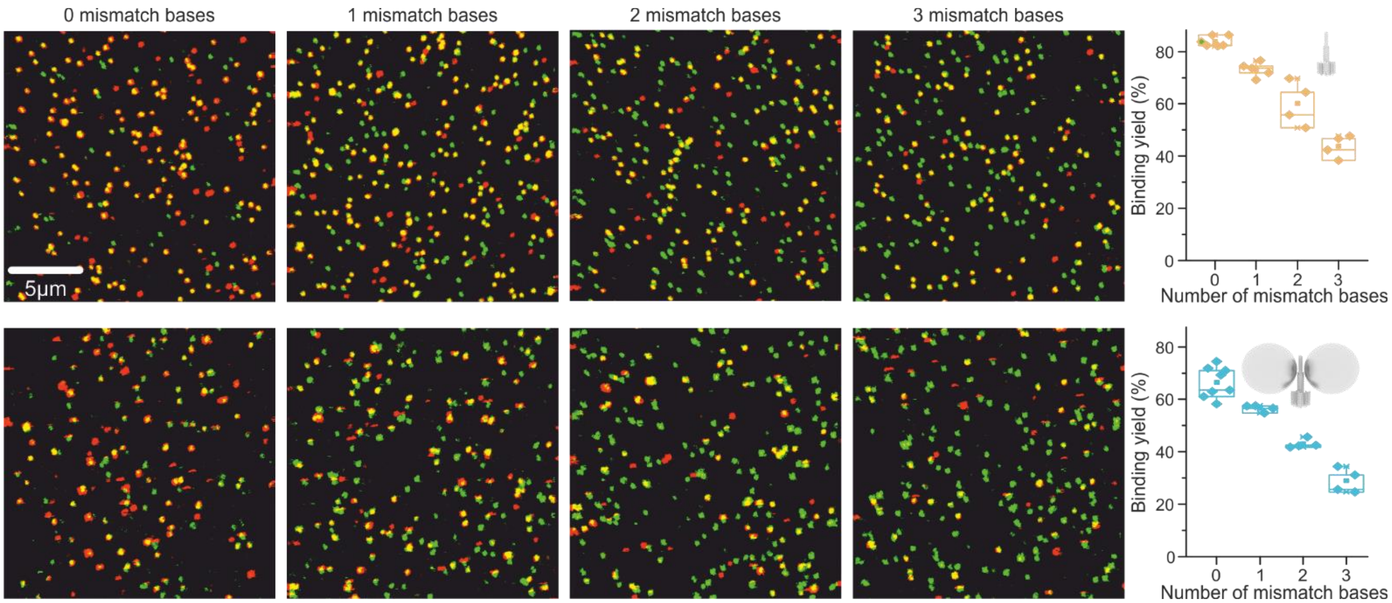


**Supplementary Figure 6. Testing specificity of binding for variations of the Oxa-48 DNA sequence: scans and binding yield for perfectly matching DNA target and targets with 1, 2, and 3 mismatches in the reference DNA nanostructure (top row) and in NACHOS (bottom row). The calculated binding yield efficiency is represented in the right panels from at least 4 different areas of each sample. The box plots show the 25/75 percentiles and the whiskers represent the 1.5*IQR (inter quartile range) values, the canter lines represent the average values. Measured at 532 nm and 639 nm with 2 µW excitation power for the reference structure and at 532 nm with 2 µW and 639 nm with 500 nW for the NACHOS structure.**

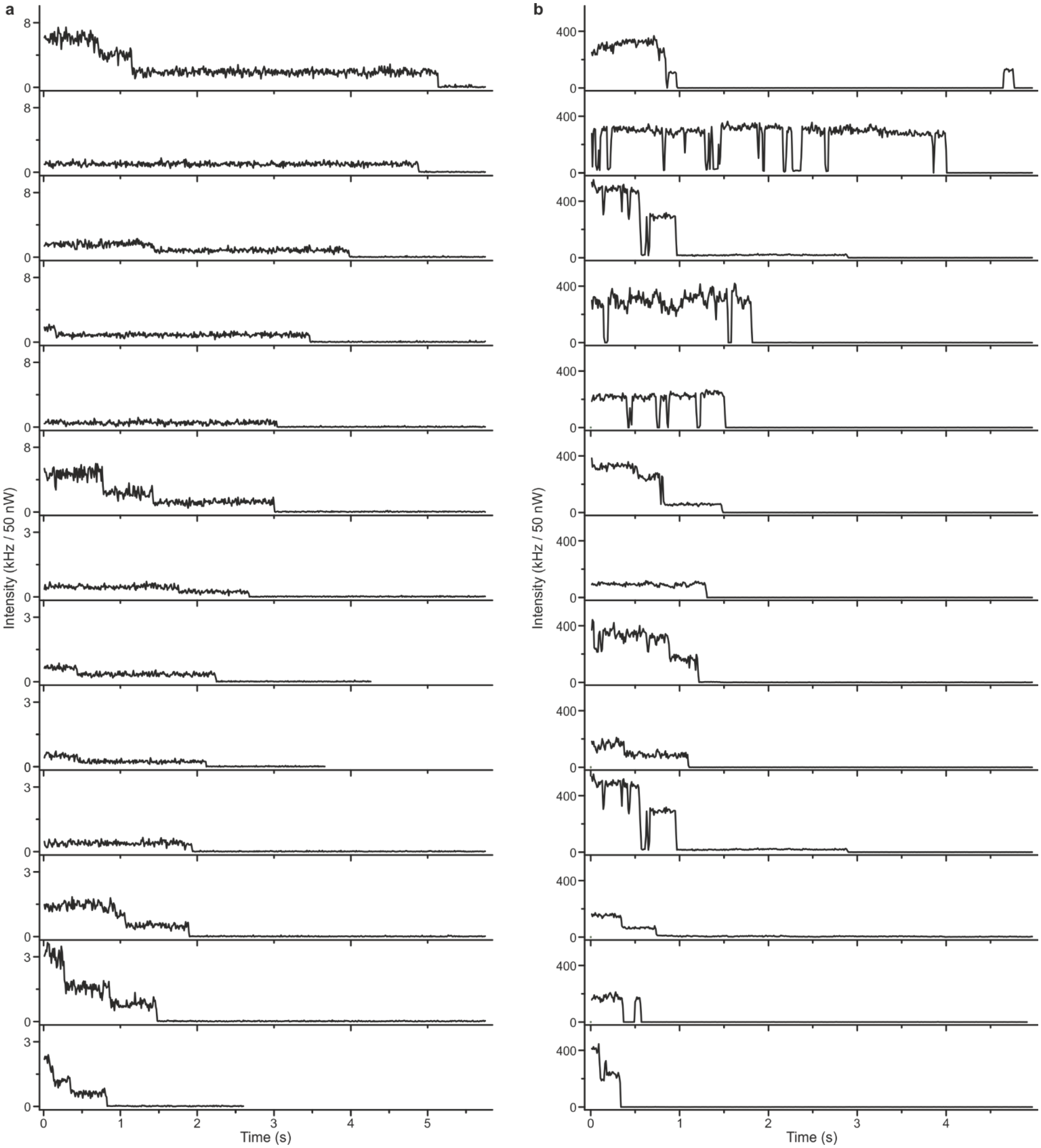


**Supplementary Figure 7. Exemplary fluorescence transients of the sandwich assay in DNA origami reference structures without nanoparticles (a) and in NACHOS (b) The samples are measured at 639 nm with 500 nW and 50 nW excitation power for panel (a) and (b), respectively, and the transients are normalized to the same excitation power.**

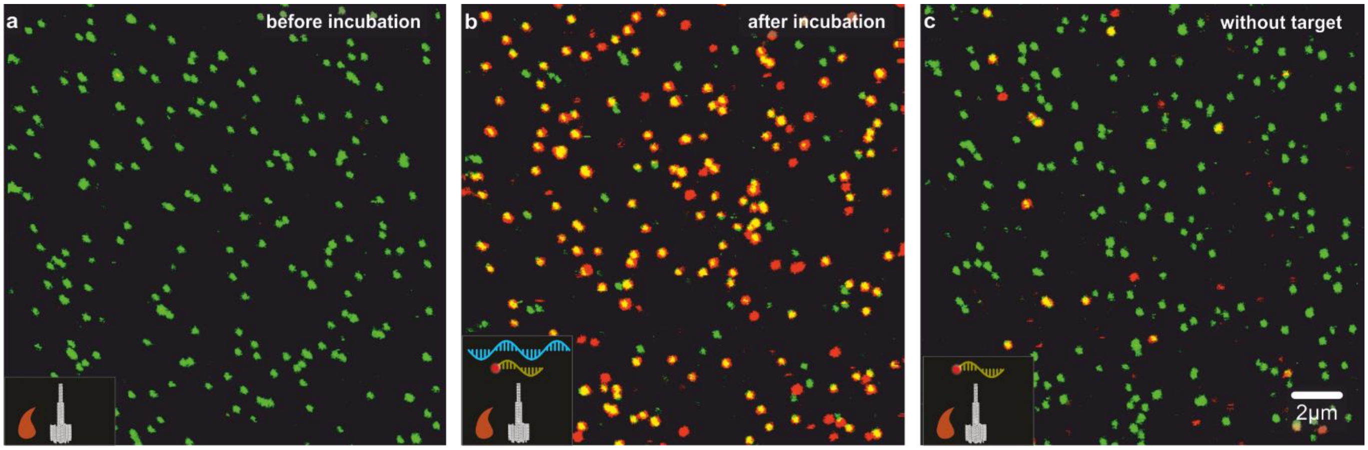


**Supplementary Figure 8. Fluorescence scans of the DNA origami reference structure (without nanoparticles) acquired in blood serum before incubation (a), after incubation with the full sandwich assay (b), and after incubation with the imager strand only (c). Measured at 532 nm and 639 nm with 2 µW excitation power. At least 20 different areas were measured for each sample.**

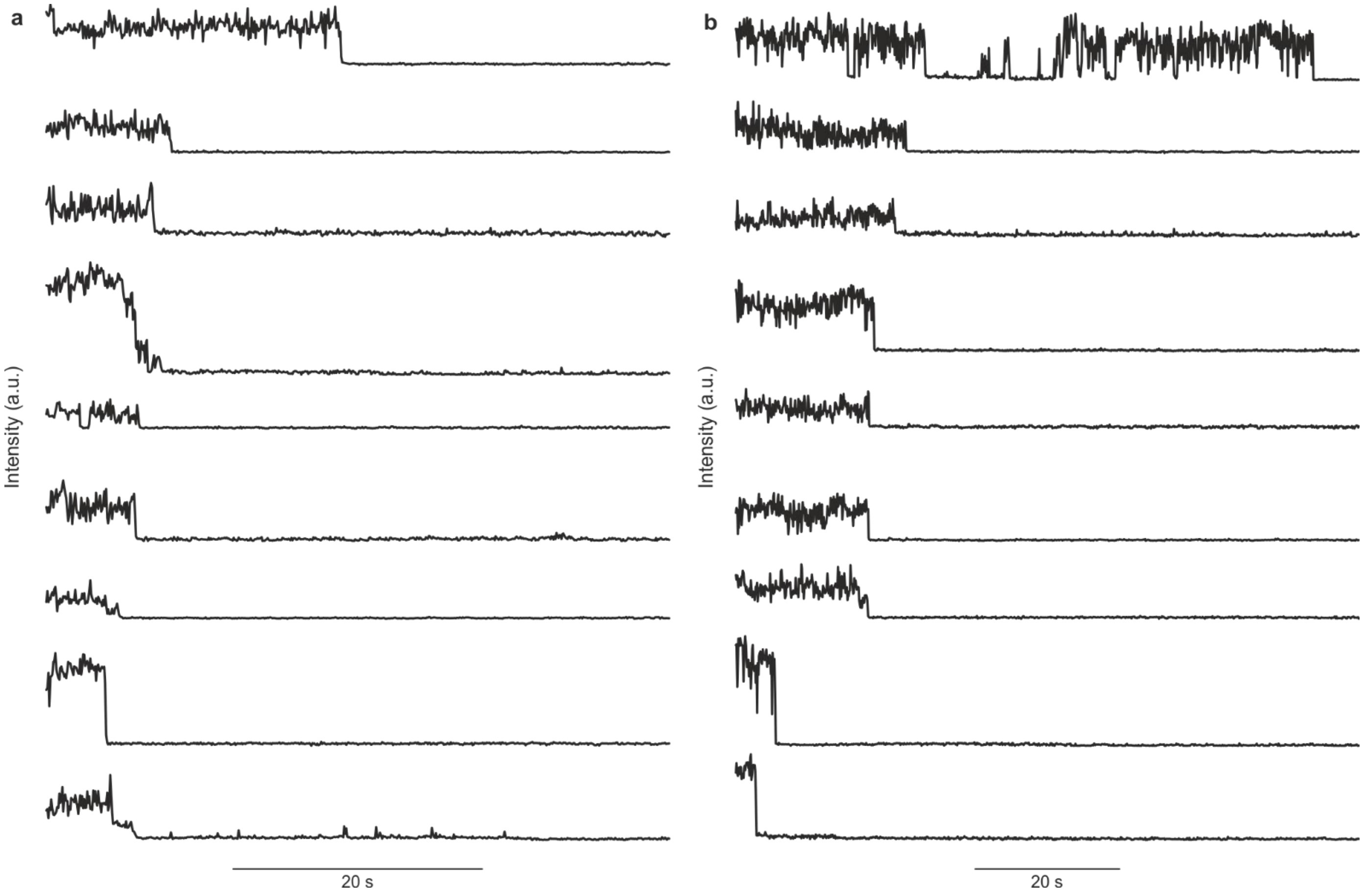


**Supplementary Figure 9. Additional fluorescence transients of single Alexa Fluor 647 dyes in NACHOS obtained from two more movies (a, b) recorded on the smartphone microscope.**

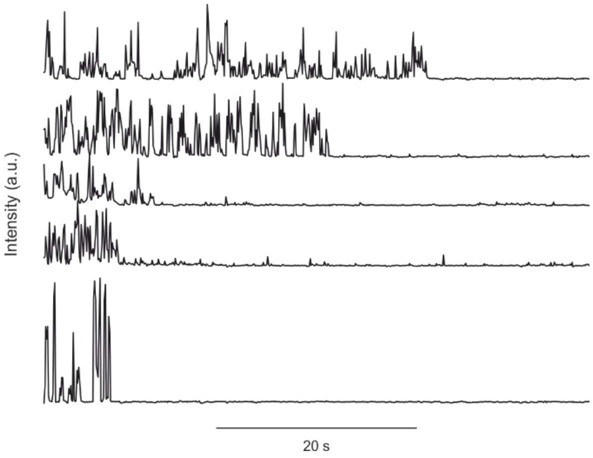


**Supplementary Figure 10. Fluorescence transients of single ATTO647N dyes in NACHOS recorded on the smartphone microscope (80 ms integration time).**

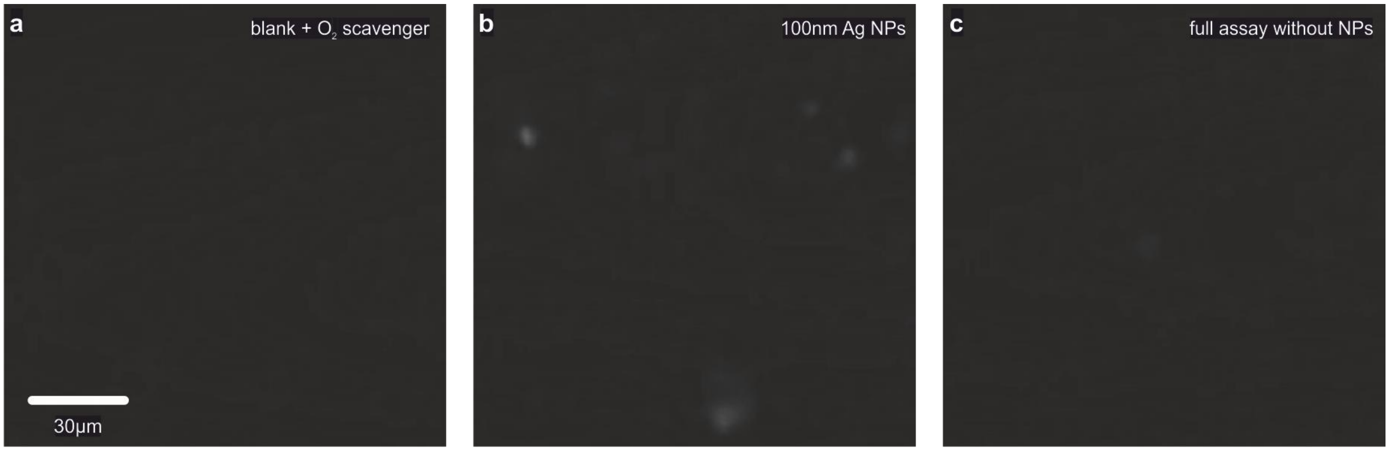


**Supplementary Figure 11. Negative controls on the smartphone (a) cleaned surface with buffer solution, (b) incubated only with 100 nm silver nanoparticles, and (c) full sandwich assay on NACHOS without silver nanoparticles in ROXS[1]. For each control measurement at least 4 movies were recorded.**

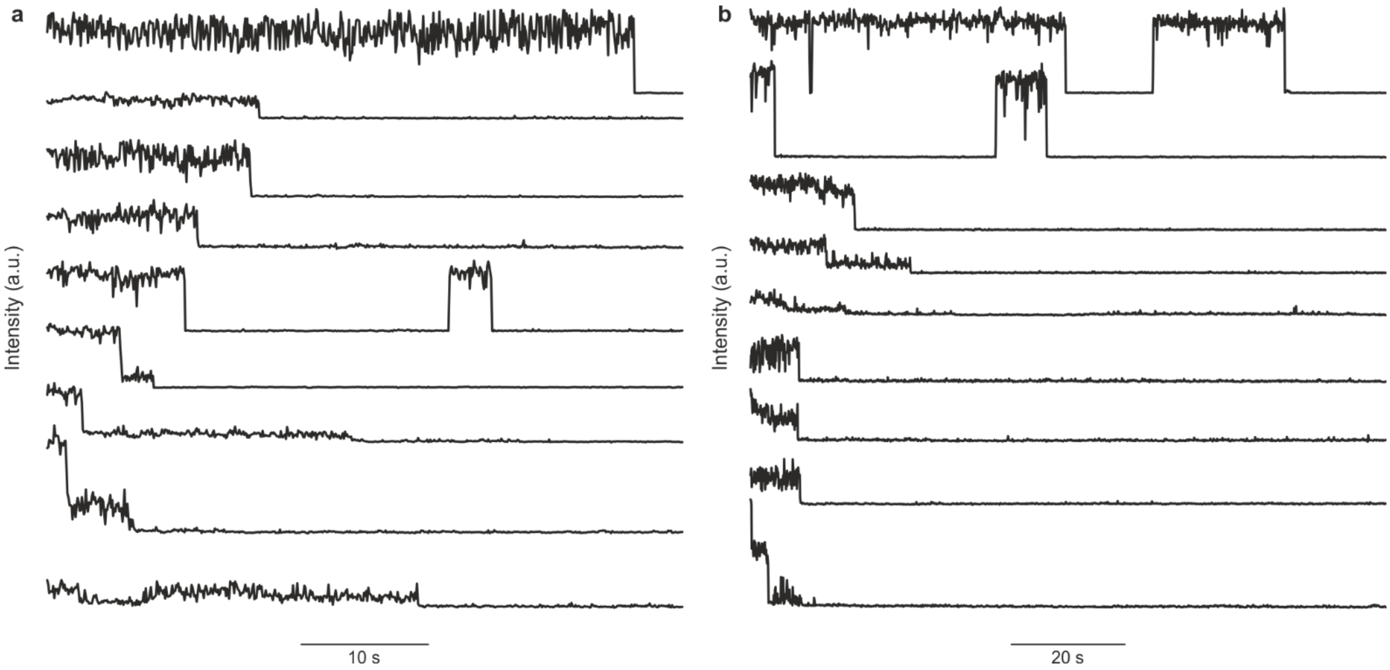


**Supplementary Figure 12. Additional fluorescence transients of the sandwich assay in NACHOS measured in buffer solution from two more movies (a, b) recorded on the smartphone microscope.**

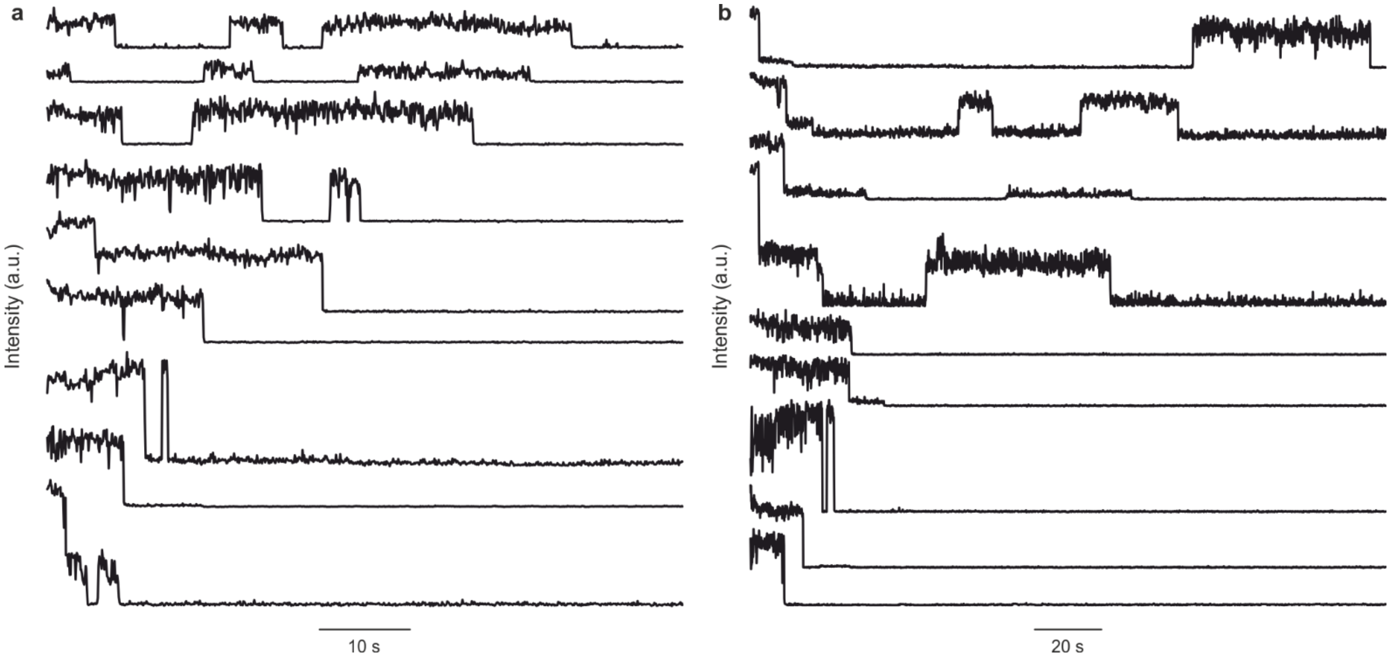


**Supplementary Figure 13. Additional fluorescence transients of the sandwich assay inside NACHOS measured in blood serum from two more movies (a, b) recorded on the smartphone microscope.**

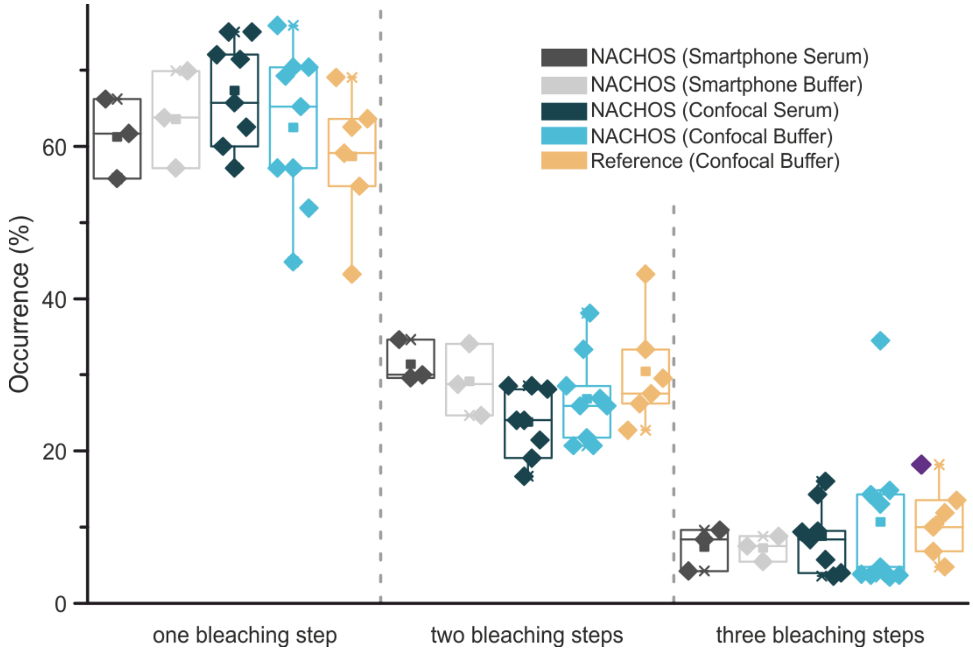


**Supplementary Figure 14. Bleaching step analysis obtained for the reference structure (orange) and for NACHOS measured on the confocal setup in buffer solution (light blue) as well as in blood serum (dark blue) (same data as shown in Fig. 2g) and for 244 traces extracted from the smartphone microscope in buffer solution (light grey) as well as in blood serum (dark grey). The box plots represent the statistics of at least 4 different areas for each sample with the 25/75 percentiles and the whiskers represent the 1.5*IQR values, the canter lines represent the average values.**

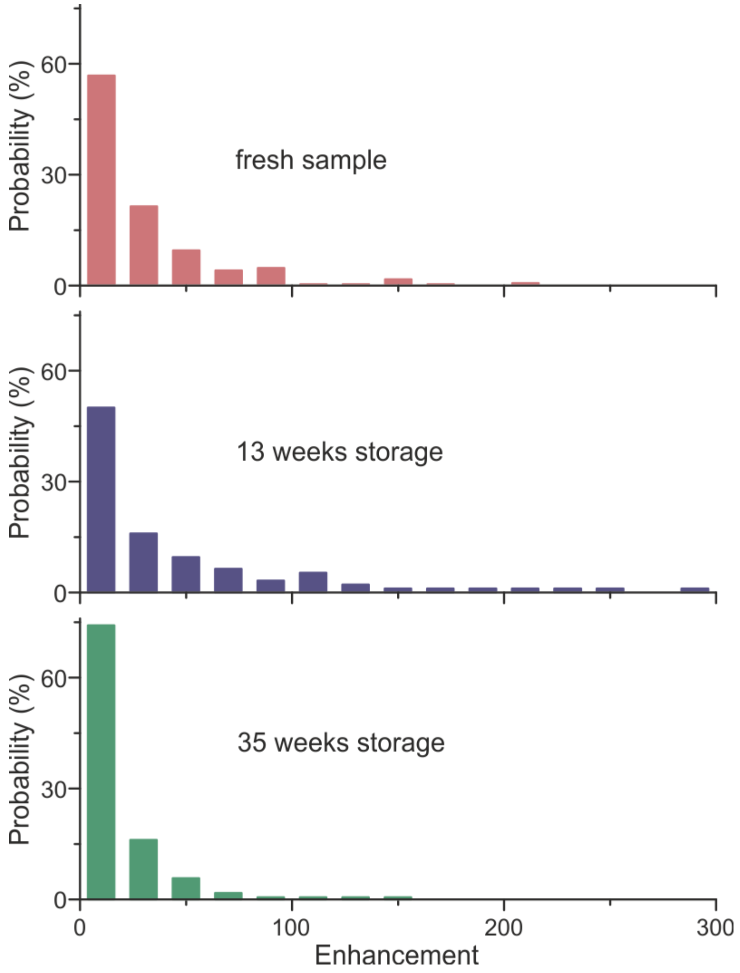


**Supplementary Figure 15. Fluorescence enhancement histograms of a single ATTO 647N dye in NACHOS of a previous design (only eight binding strands of $A_{25}$ for nanoparticles, $T_{25}$-SH used for nanoparticles functionalization). No difference between the fresh sample (red, 294 molecules measured) and the sample measured after 13 weeks (blue, 94 molecules) were observed. Slight changes are visible for the sample measured after ~35 weeks (green, 174 molecules). The sample (Lab-Tek™ II-chambers with TE buffer containing 14 mM $MgCl_2$ was stored at 4 °C and care was taken to avoid drying of the sample. At least 5 areas were measured for each time point.**

**Supplementary Note 1. Discussion pertaining the costs of the smartphone microscope**

**Price list of the current smartphone microscope**

| Name of the component | Price |
|---|---|
| Excitation source: Integrated Optics 0638L-11A (Lithuania) Laser incl. power bank and cooling system | 1892 € |
| Smartphone: Huawei P20 (China) | 439 € |
| Objective Lens: UCTRONICS LS-40166 (USA) | ~8 € |
| Filter: Semrock Inc. BrightLine HC 731/137 (USA) | 472 € |
| Focussing lens: Thorlabs Inc. AC254-050-A-ML (USA) | 114 € |
| Sample positioner: 3× Thorlabs Inc. MT1/M (USA) | 3× 341 € = 1023 € |
| Laser positioning: Thorlabs Inc. Optomechanical Components | 218 € |
| **Total sum:** | ~4200 € |

**Estimated pricelist of future smartphone microscopes**

X and Y positioners can be omitted or substituted by cheaper ones since the accuracy is not needed inside the microscope.
Large scale production of the filters with a customize size can reduce the price by at least one order of magnitude, as a currently used standard commercially available filter is big enough to provide material for over 10 filters for smartphone microscopes.
Focussing lens does not have to be an achromatic one, i.e. price reduction to ~30 % of original price possible.
Smartphone can be cheaper especially if the smartphone is specialized for camera performance -> price reduction ~50 % possible. We also note that the current smartphone was purchased in early 2019 and the current value of the same smartphone is substantially lower right now. The power density in the current configuration is set to ~ 600 $\mu Wcm^{-1}$. Due to the high signal-to-background ratio we estimate that a lower power density would also be enough to make NACHOS visible on the smartphone microscope. This can be easily achieved by a high-power LED and an excitation filter to narrow down the excitation spectrum. This kind of LED in combination with a high-end excitation filter in suitable size can reduce the price to ~ 200 €.

| Name of the component | Estimated price |
|---|---|
| Excitation source: e.g. Mouser, 897-LZ110R1020000 incl. power bank and bandpass filter Chroma 620/60 ET (USA) | 200 € |
| Smartphone | 220 € |
| Objective Lens: UCTRONICS LS-40166 (USA) | ~8 € |
| Filter: Semrock Inc. BrightLine HC 731/137 (USA) | 45 € |
| Focussing lens | 37 € |
| Sample positioner (Z axis): Thorlabs Inc. MT1/M (USA) | 341 € |
| Laser positioning: Thorlabs Inc. Optomechanical Components | 218 € |
| **Total sum:** | **~1000 €** |

Additional discounts of at least 30 % can be expected for large scale purchase of the single components -> final price < 700 € possible.

**Supplementary Note 2. Discussion pertaining the costs per sample for the diagnostic assay on a smartphone microscope.**

To estimate the price of materials and consumables used for the preparation of one sample, the prices stated in recent bills were used and then divided by the amount of samples that can be prepared from the ordered product.

| **Name of the product** | **Price, €** | **Total volume/ mass/ number of pieces of the product** | **Volume/ mass/ number of pieces used for one sample** | **Estimated price for one sample, €** |
|---|---|---|---|---|
| **Coverslip 22 mm × 22 mm** | 21.5 | 200 pieces | 1 | 0.11 |
| **Microscope slide** | 2.95 | 50 pieces | 1 | 0.06 |
| **BSA-biotin**[a] | 158 | 10 mg | 0.075 mg | 1.19 |
| **NeutrAvidin**[a] | 204 | 10 mg | 0.03 mg | 0.67 |
| **Unmodified DNA staple strands**[b] | 1200 | 200 staples, 100 µl each | 1 set per 30,000 samples | 0.04 |
| **Modified staples**[b] | 300 | 6 biotin strands, 100 uL each | 1 set per 180,000 samples | 0.02 |
| **Scaffold***[b] | 125 | 0.5 ml of 100 nM | 1 bottle for 12,000 samples | 0.01 |
| **Amicon filter**[b] | 407 | 96 | 1 filter per 600 samples | 0.07 |
| **100 nm BioPure Silver Nanospheres (nanoComposix, USA)**[a] | 215 | 1 ml | 1 bottle for 250 samples | 0.86 |
| **Thiolated oligos**[a] | 170 | 50 bottles of 1 nmol | 1 bottle for 5 samples | 0.68 |
| **Imager strand**[a] | 150 | 100 uL of 100 µM | 1 bottle for 17000 samples | 0.09 |
| **Other (buffers, silicon form, electricity, water...)**[c] | | | | < 1 |
| | | | **Total:** | **~ 4.8 €** |

[a] Calculation is done based on concentrations given in materials and methods section

[b] One preparation of the DNA origami stock (~20 µl of ~ 50 nM) requires 18 uL of pool from unmodified staples, 2 uL of pool from modified staples, 25 uL of the scaffold, and 1 Amicon filter. To prepare one sample for the smartphone measurements 150 µl of 10 pM is required, that is, one DNA origami stock is enough to prepare > 600 samples for the smartphone measurements.

* For the estimation, the price of commercially available scaffold was used. Produced in-house scaffold will yield a lower price.

[c] Estimation takes into account costs that are hard to estimate and handling mistakes

The prices can be further reduced by larger scale purchases.

**Supplementary References**


1. Vogelsang, J. et al. A Reducing and Oxidizing System Minimizes Photobleaching and Blinking of Fluorescent Dyes. *Angewandte Chemie International Edition* **47**, 5465-5469 (2008).
