# Supplementary material for: Addressable nanoantennas with cleared hotspots for single-molecule detection on a portable smartphone microscope
Source: Nat Commun. 2021 Feb 11;12:950. doi: 10.1038/s41467-021-21238-9 (PMC7878865; doi:10.1038/s41467-021-21238-9)
Supplement: Supplementary file 3 — Description of Additional Supplementary Files [file 41467_2021_21238_MOESM3_ESM.pdf]

Description of Additional Supplementary Files

File Name: Supplementary Movie 1
Description: Single-molecule video of Alexa Fluor 647 in the hotspot of NACHOS obtained with the smartphone microscope

File Name: Supplementary Movie 2
Description: Single-molecule video of Alexa Fluor 647 the hotspot of NACHOS obtained with the smartphone microscope

File Name: Supplementary Movie 3
Description: Single-molecule video of Alexa Fluor 647 the hotspot of NACHOS obtained with the smartphone microscope

File Name: Supplementary Movie 4
Description: Single-molecule video of ATTO 647N the hotspot of NACHOS obtained with the smartphone microscope

File Name: Supplementary Movie 5
Description: Sandwich assay inside NACHOS detecting a single DNA molecule measured in buffer recorded on the smartphone microscope

File Name: Supplementary Movie 6
Description: Sandwich assay inside NACHOS detecting a single DNA molecule measured in buffer recorded on the smartphone microscope

File Name: Supplementary Movie 7
Description: Sandwich assay inside NACHOS detecting a single DNA molecule measured in buffer recorded on the smartphone microscope

File Name: Supplementary Movie 8
Description: Sandwich assay inside NACHOS detecting a single DNA molecule measured in blood serum recorded on the smartphone microscope

File Name: Supplementary Movie 9
Description: Sandwich assay inside NACHOS detecting a single DNA molecule measured in blood serum recorded on the smartphone microscope

File Name: Supplementary Movie 10
Description: Sandwich assay inside NACHOS detecting a single DNA molecule measured in blood serum recorded on the smartphone microscope
