# Supplementary material for: Addressable nanoantennas with cleared hotspots for single-molecule detection on a portable smartphone microscope
Source: Nat Commun. 2021 Feb 11;12:950. doi: 10.1038/s41467-021-21238-9 (PMC7878865; doi:10.1038/s41467-021-21238-9)
Supplement: Supplementary file 14 — Reporting Summary [file 41467_2021_21238_MOESM14_ESM.pdf]

nature research

Corresponding author(s): Kateryna Trofymchuk, Viktorija Glembockyte, Guillermo P. Acuna, Philip Tinnefeld

Last updated by author(s): Dec 22, 2020

Reporting Summary

Nature Research wishes to improve the reproducibility of the work that we publish. This form provides structure for consistency and transparency in reporting. For further information on Nature Research policies, see our Editorial Policies and the Editorial Policy Checklist.

Statistics

For all statistical analyses, confirm that the following items are present in the figure legend, table legend, main text, or Methods section.

n/a	Confirmed	
☐	☒	The exact sample size (n) for each experimental group/condition, given as a discrete number and unit of measurement
☐	☒	A statement on whether measurements were taken from distinct samples or whether the same sample was measured repeatedly
☒	☐	The statistical test(s) used AND whether they are one- or two-sided<br>*Only common tests should be described solely by name; describe more complex techniques in the Methods section.*
☒	☐	A description of all covariates tested
☒	☐	A description of any assumptions or corrections, such as tests of normality and adjustment for multiple comparisons
☐	☒	A full description of the statistical parameters including central tendency (e.g. means) or other basic estimates (e.g. regression coefficient) AND variation (e.g. standard deviation) or associated estimates of uncertainty (e.g. confidence intervals)
☒	☐	For null hypothesis testing, the test statistic (e.g. F, t, r) with confidence intervals, effect sizes, degrees of freedom and P value noted<br>*Give P values as exact values whenever suitable.*
☒	☐	For Bayesian analysis, information on the choice of priors and Markov chain Monte Carlo settings
☒	☐	For hierarchical and complex designs, identification of the appropriate level for tests and full reporting of outcomes
☒	☐	Estimates of effect sizes (e.g. Cohen's d, Pearson's r), indicating how they were calculated

Our web collection on statistics for biologists contains articles on many of the points above.

Software and code

Policy information about availability of computer code

Data collection	A custom-made LabVIEW 2012 software (National Instruments, USA) for the confocal measurements. FreeDCam v4.2 application for the smartphone microscope measurements.
Data analysis	A custom-made LabVIEW 2012 software (National Instruments, USA), ImageJ v1.52p (FIJI) with FFMPEG (v4.1-1.4.4) plugin and a home written macro (available at a public repository Zenodo (10.5281/zenodo.4384169)), OriginPro2016.

For manuscripts utilizing custom algorithms or software that are central to the research but not yet described in published literature, software must be made available to editors and reviewers. We strongly encourage code deposition in a community repository (e.g. GitHub). See the Nature Research guidelines for submitting code & software for further information.

Data

Policy information about availability of data

All manuscripts must include a data availability statement. This statement should provide the following information, where applicable:

- Accession codes, unique identifiers, or web links for publicly available datasets
- A list of figures that have associated raw data
- A description of any restrictions on data availability

The raw data acquired in this study are available in a public Zenodo repository (10.5281/zenodo.4384169). This includes TEM images, raw and analyzed confocal data, raw movies acquired on the smartphone device, as well as the caDNAno file for the DNA origami nanostructure reported in this work. Further information is available from the authors upon request.

Field-specific reporting

Please select the one below that is the best fit for your research. If you are not sure, read the appropriate sections before making your selection.

☒ Life sciences ☐ Behavioural & social sciences ☐ Ecological, evolutionary & environmental sciences

For a reference copy of the document with all sections, see nature.com/documents/nr-reporting-summary-flat.pdf

Life sciences study design

All studies must disclose on these points even when the disclosure is negative.

Sample size	Number of analyzed single molecule-transients or single-molecule spots on confocal scans and smartphone images determine the sample sizes. It was ensured that the sample sizes were equal or greater to sample sizes reported in literature to enable comparison.
Data exclusions	Transients that did not show single-molecule behavior and confocal spots with obviously not circular PSF (e.g. due to DNA origami aggregation) were excluded from analysis.
Replication	Every graph in the manuscript represents data analyzed from at least 150 molecules from one experiment, however, all measurements were repeated at least three times on independently prepared samples to confirm the reproducibility of the findings. Other datasets are available upon request.
Randomization	The studies are focused on the imaging techniques and do not imply randomization. We analysed every dataset in the same way, which is why there was no requirement for randomization in our case.
Blinding	The studies are focused on the imaging techniques and do not require blinding. Furthermore, we could not employ blind studies since non-enhanced and enhanced samples can be clearly distinguished already during the measurement.

Reporting for specific materials, systems and methods

We require information from authors about some types of materials, experimental systems and methods used in many studies. Here, indicate whether each material, system or method listed is relevant to your study. If you are not sure if a list item applies to your research, read the appropriate section before selecting a response.

Materials & experimental systems

n/a	Involved in the study	
☒	☐	Antibodies
☒	☐	Eukaryotic cell lines
☒	☐	Palaeontology and archaeology
☒	☐	Animals and other organisms
☒	☐	Human research participants
☒	☐	Clinical data
☒	☐	Dual use research of concern

Methods

n/a	Involved in the study	
☒	☐	ChIP-seq
☒	☐	Flow cytometry
☒	☐	MRI-based neuroimaging
